# New Radioligands for Describing the Molecular Pharmacology of MT_1_ and MT_2_ Melatonin Receptors

**DOI:** 10.3390/ijms14058948

**Published:** 2013-04-25

**Authors:** Céline Legros, Ulrich Matthey, Teresa Grelak, Sandrine Pedragona-Moreau, Werner Hassler, Saïd Yous, Emmanuel Thomas, Franck Suzenet, Benoît Folleas, François Lefoulon, Pascal Berthelot, Daniel-Henri Caignard, Gérald Guillaumet, Philippe Delagrange, Jean-Louis Brayer, Olivier Nosjean, Jean A. Boutin

**Affiliations:** 1 BPMC, Institut de Recherches SERVIER, 125 chemin de Ronde, Croissy-sur-Seine 78290, France; E-Mails: celine.legros@fr.netgrs.com (C.L.); olivier.nosjean@fr.netgrs.com (O.N.); 2 Celerion Switzerland AG Allmendstrasse 32, Fehraltorf CH-8320, Switzerland; E-Mails: Ulrich.matthey@celerion.com (U.M.); teresa.grelak@celerion.com (T.G.); 3 Technologie SERVIER, 27 rue Vignat, Orléans 45000, France; E-Mails: sandrine.moreau@fr.netgrs.com (S.P.-M.); francois.lefoulon@fr.netgrs.com (F.L.); 4 ANAWA Trading SA, Unterdorfstrasse 21, Wangen CH-8602, Switzerland; E-Mail: hassler@anawa.ch; 5 Université Lille Nord de France, F-59000 Lille, France & UDSL, EA GRIIOT, UFR Pharmacie, Lille F-59000, France; E-Mail: said.yous@univ-lille2.fr (S.Y.); pascal.berthelot@univ-lille2.fr (P.B.); 6 DIVERCHIM, 6 Rue du Noyer, Roissy 95700, France; E-Mails: emmanuel.thomas@diverchim.com (E.T.); benoit.folleas@diverchim.com (B.F.); jean-louis.brayer@diverchim.fr (J.-L.B.); 7 Institut de Chimie Organique et Analytique, UMR CNRS 7311, Université d’Orléans, rue de Chartres, Orléans 45067, France; E-Mails: franck.suzenet@univ-orleans.fr (F.S.); gerald.guillaumet@univ-orleans.fr (G.G.); 8 Unité de Recherches et Découvertes en Neurosciences, Institut de Recherches SERVIER, 125 chemin de Ronde, Croissy-sur-Seine 78290, France; E-Mails: daniel-henri.caignard@fr.netgrs.com (D.-H.C.); philippe.delagrange@fr.netgrs.com (P.D.)

**Keywords:** melatonin receptors, 2-iodomelatonin, alternative radioligands, synthesis, radiolabeling

## Abstract

Melatonin receptors have been studied for several decades. The low expression of the receptors in tissues led the scientific community to find a substitute for the natural hormone melatonin, the agonist 2-[^125^I]-iodomelatonin. Using the agonist, several hundreds of studies were conducted, including the discovery of agonists and antagonists for the receptors and minute details about their molecular behavior. Recently, we attempted to expand the panel of radioligands available for studying the melatonin receptors by using the newly discovered compounds SD6, DIV880, and S70254. These compounds were characterized for their affinities to the hMT_1_ and hMT_2_ recombinant receptors and their functionality in the classical GTPγS system. SD6 is a full agonist, equilibrated between the receptor isoforms, whereas S70254 and DIV880 are only partial MT_2_ agonists, with K_i_ in the low nanomolar range while they have no affinity to MT_1_ receptors. These new tools will hopefully allow for additions to the current body of information on the native localization of the receptor isoforms in tissues.

## 1. Introduction

Melatonin is a neurohormone produced by the pineal gland at night [1,2] and is thought to control circadian rhythm. The activity of melatonin is relayed primarily by the seven transmembrane G protein-coupled receptors MT_1_ and MT_2_ [3]. The pharmacological actions of melatonin at higher concentrations (μM and above) are thought to be mediated by other protein targets, such as QR2 [3,4]. Before these receptors were cloned in the late 1990s [5,6], the measurement of melatonin binding to membranes derived from any animal organ was difficult because the level of expression is naturally very low for these receptors, and the available radioligand ([^3^H]-melatonin) could not be synthesized with enough specific activity.

In 1984, a Finnish group described the use of a more sensible tool for binding studies: 2-[^125^I]-iodomelatonin ([^125^I]-2IMLT), a strongly labeled super-agonist of the receptors [7,8]. Almost immediately, all experiments reported in the literature used this tool, and the labeled natural hormone ([^3^H]-melatonin) was not used again until a later, complete study [9]. To determine whether other tools for molecular pharmacology studies of melatonin are useful, particularly regarding the agonist nature of this ligand (2-iodomelatonin, 2IMLT), we sought an alternative to [^125^I]-2IMLT by screening our compounds to identify antagonist(s) or partial agonists (30% and below) for any or both receptor isoforms. The aim was to broaden the panel of available tools for studying these receptors [3,10]. By conducting several series of high throughput screening HTS campaigns [11,12], we found a MT_2_-specific partial agonist, DIV880. The K_i_ of this compound is 2 logs less potent with MT_1_ than MT_2_. As a first step, we synthesized the precursors of each ligand, iodinated them, and assessed their binding characteristics with recombinant human MT_1_ and MT_2_ receptors.

Over the last few years, our search for new ligands has been driven mainly by the addition of more molecular tools to the available panel of molecules used to study melatonin receptors, such as ligands specific to MT_1_ or MT_2_ and antagonist(s) of the melatonin receptors. Such a discovery would help broaden our understanding of this system for which almost no molecules have been reported [13]. It thus follows that such a molecule would then be labeled in order to obtain new ligands.

## 2. Results and Discussion

**Figure 1:**
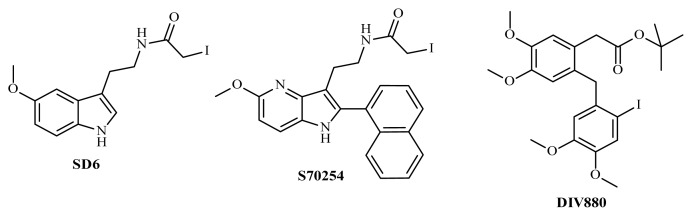
Structures of the new ligands used in the present study.

SD6 and S70254 were the products of a rational exploration of the basic features of melatonin analogs and are structurally related to the natural hormone (Figure 1). The influence of iodine atoms on the core structures was studied systematically, leading to interesting compounds that can be used to complete the set of labeled compounds for describing the molecular pharmacology of melatonin receptor(s). For DIV880, the process was a little different. The compound is an iodinated analog of a bromo-compound that resulted from a large screening process, similar to the compounds described elsewhere [11,12]. This compound was specific to MT_2_, with a pK_i_ 2 logs “better” for MT_2_ than for MT_1_. Therefore, the iodine equivalent was synthesized, leading to its possible use as a specific probe for MT_2_.

Prior to radiolabeling the compounds, we characterized the cold compounds for recombinant human MT_1_ and MT_2_ receptors and compared them to 2IMLT (Table 1). 2IMLT and SD6 shared similar properties, particularly similar affinities for the receptors in the low nanomolar range, similar potency in the GTPγS assay, with full agonistic effects for both receptors (E_max_ > 100%), and almost similar pEC_50_ for both receptors (with a minor discrepancy for MT_1_) in a TR-FRET cAMP assay (E_max_ ~100%). S70254 was a poor MT_1_ ligand, in the micromolar range (pK_i_ = 6.18), but a good MT_2_ ligand (pK_i_ = 8.73). The functionality of S72054 remained measurable but poor for MT_1_, but was a partial agonist for MT_2_ (43%) in the GTPγS assay and a less partial agonist (73%) in the TR-FRET cAMP assay. Finally, for DIV880, the affinity for MT_1_ remained in the micromolar range, but it was in the 10 nM range for MT_2_. Similarly, the functional effect of this compound was impossible to record using both functional assays with MT_1_, but the compound behaved as a partial agonist (67%) of MT_2_ in the GTPγS assay and full agonist (97%) of MT_2_ in the TR-FRET cAMP assay.

Overall, we identified two MT_2_-specific ligands, S70254 and DIV880, and two aspecific, full agonistic compounds, SD6 and iodomelatonin, for both receptors.

**Figure 2:**
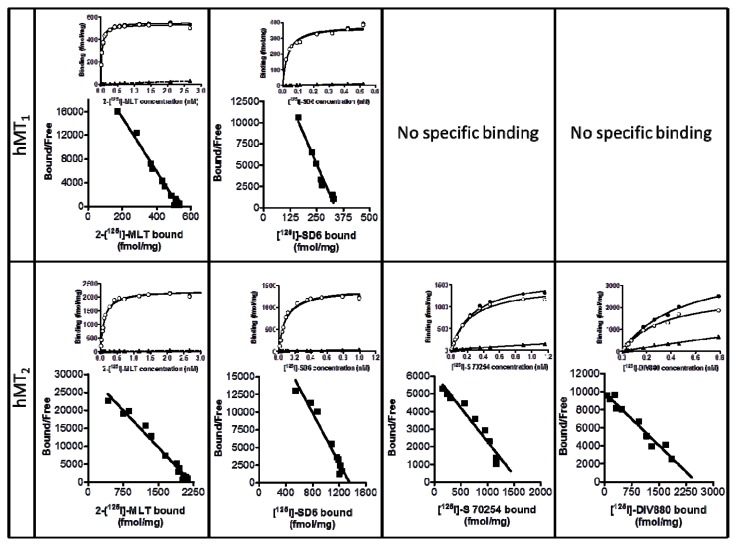
Saturation and Scatchard regression of the four radioligands of human recombinant MT_1_ and MT_2_ receptors: 2-[^125^I]-iodomelatonin, [^125^I]-SD6, [^125^I]-S70254, and [^125^I]-DIV880. The curves are individual results representative of at least three independent experiments. Full circles, total binding; open circles, specific binding; and close triangles, non-specific binding.

We proceeded to label the compounds with [^125^I], and characterized the ligands for both recombinant receptors. The compounds behaved properly in the binding assays after a rapid assessment of the experimental conditions. Figure 2 clearly indicates standard behavior comparable to [^125^I]-2IMLT for the three compounds. As expected, the four compounds bound MT_2_, but only [^125^I]-2IMLT and [^125^I]-SD6 bound MT_1_. The pK_b_ and B_max_ values are given in Table 2.

Interestingly, the compounds did not label the same amount of MT_1_ receptor (SD6: 276 fmol/mg of protein; [^125^I]-2IMLT: 688 fmol/mg of protein), strongly suggesting that the compounds “see” different states of the receptor. This feature needs to be explored further and the corresponding experiments are ongoing in our laboratory.

The present work offers the first alternative to the classical ligands [^125^I]-2IMLT and [^3^H]-melatonin. [^125^I]-2IMLT has been monopolizing the melatonin binding field since it was first described by Vakkuri *et al.* [7], and [^3^H]-melatonin was completely described in 2000 [9] and rarely used then after. [^125^I]-S70254 and [^125^I]-DIV880 will permit the first specific investigation of the hMT2 receptor, as well as cellular systems overexpressing MT receptors and tissue samples (e.g., binding and autoradiography).

## 3. Experimental Section

### 3.1. Reagents and Ligands

 [^125^I]-SD6, [^125^I]-S70254, and [^125^I]-DIV880 were custom-made by ANAWA Trading SA (Wangen/Zürich, Switzerland). The specific activity was 2,175 Ci/mmol for [^125^I]-SD6, [^125^I]-S70254, and [^125^I]-DIV880. [^125^I]-2IMLT (specific activity 2,200 Ci/mmol) was purchased from Perkin Elmer (Boston, MA, USA). Melatonin and other reagents were obtained from Sigma (St. Louis, MO, USA). Melatonin was dissolved in DMSO at a stock concentration of 10 mM and stored at −20 °C.

### 3.2. Membrane Preparation

CHO-K1 cell lines stably expressing the human MT_1_ or MT_2_ receptors [14] were grown to confluence, harvested in PBS buffer (Gibco, Invitrogen, Saint-Aubin, France) containing 5 mM EDTA, and centrifuged at 1,000 g for 20 min (4 °C). The resulting pellet was suspended in 5 mM Tris/HCl (pH 7.4) containing 2 mM EDTA and homogenized using Kinematicapolytron. The homogenate was then centrifuged (20,000 g, 30 min, 4 °C) and the resulting pellet suspended in 75 mM Tris/HCl (pH 7.4) containing 2 mM EDTA and 12.5 mM MgCl_2_. Protein content was determined according to Bradford [15] using the Bio-Rad kit (Bio-Rad SA, Ivry-sur-Seine, France). Aliquots of membrane preparations were stored in re-suspension buffer (75 mM Tris/HCl pH 7.4, 2 mM EDTA, 12.5 mM MgCl_2_) at −80 °C until use.

### 3.3. Membrane Binding Assays

#### 3.3.1. 2-[^125^I]-iodomelatonin and [^35^S]-GTPγS Binding Assays

The assays were described previously [16]. Briefly, for competition experiments in CHO cells, the membranes were incubated in 250 μL binding buffer (50 mM Tris/HCl pH 7.4, 5 mM MgCl_2_) containing 20 pM [^125^I]-2IMLT for 2 h at 37 °C. The results were expressed as the inhibition constant Ki, taking into account the concentration of radioligand used in each experiment. Non-specific binding was defined using 10 μM melatonin. The reaction was stopped by rapid filtration through GF/B unifilters, followed by three successive washes with ice-cold buffer. The data were analyzed using the program PRISM (GraphPad Software Inc., San Diego, CA, USA). K_i_ was calculated according to the Cheng–Prussof Equation: K_i_ = IC_50_/[1 + (L/K_d_)], where IC_50_ is the half maximal inhibitory concentration and L is the concentration of [^125^I]-2IMLT [17].

For the [^35^S]-GTPγS binding assay, the membranes and compounds were diluted in the binding buffer (20 mM Hepes pH 7.4, 100 mM NaCl, 3 mM MgCl_2_, 3 μM GDP) in the presence of 20 μg/mL saponin in order to enhance the agonist-induced stimulation [16]. Incubation was started by adding 0.1 nM [^35^S]-GTPγS to the membranes and ligands in a final volume of 250 μL and allowed to continue for 60 min at room temperature. Non-specific binding was assessed using non-radiolabeled GTPγS (10 μM). Reactions were stopped by rapid filtration through GF/B unifilters pre-soaked with distilled water, followed by three successive washes with ice-cold buffer. The data were analyzed using the program PRISM to yield the half maximal effective concentration (EC_50_) and maximal effect (E_max_) expressed as a percentage of that observed with melatonin (1 μM = 100%). pEC_50_ was calculated as pEC_50_ = −log(EC_50_).

#### 3.3.2. New Ligand Binding Assays

The assays were performed in 96-well plates in 250 μL binding buffer (50 mM Tris/HCl pH 7.4, 5 mM MgCl_2_, 1 mM EDTA, plus BSA 0.1% for [^125^I]-DIV880). The membranes, hMT_1_ and hMT_2_, were used at a final concentration of 30 μg of proteins/mL for all radioactive compounds. For all protocols, the reaction was stopped by rapid filtration through GF/B unifilters (PEI 0.1% treated for [^125^I]-DIV880), followed by three successive washes with ice-cold buffer (50 mM Tris/HCl, pH 7.4). For saturation experiments with CHO-K1-hMT_1_ and hMT_2_, the membranes were incubated for 2 h at 37 °C, the time to reach the equilibrium determined by the mass-action law, in binding buffer containing 0.01–2 nM of an iodinated compound: 2-[^125^I]-2IMLT, [^125^I]-DIV880, [^125^I]-S70254, and [^125^I]-SD6.

The data were analyzed using the program PRISM (GraphPad Software Inc., San Diego, CA, USA). For the saturation assay, the binding site density (B_max_) and dissociation constant for the radioligand (K_d_) were calculated according to the Scatchard method.

### 3.4. HTRF cAMP Assay

Cellular cAMP production was measured using cAMP dynamic HTRF kits (Cisbio Bioassays, Bedford, MA, USA) according to the manufacturer’s instructions. CHO-K1 cells stably expressing the hMT_1_ or hMT_2_ receptor were grown to confluence, harvested in PBS buffer containing 5 mM EDTA, and centrifuged at 100× *g* for 10 min (4 °C). The cell pellet was re-suspended in 0.5 mM HAMF12 IBMX at a concentration of 2 million cells/mL. Incubation was started by adding 5 μM forskolin (15 μL/well) to the cells (30,000 cells/well) and compounds (15 μL/well, DMSO 1.7%) in a final volume of 60 μL, and allowed to continue for 20 min at 37 °C. Next, 15 μL of cAMP-d2 conjugate and 15 μL of anti-cAMP-EuK conjugate in lysis buffer were incubated for 30 min at room temperature. The fluorescence intensity was measured at 340 nm excitation and 665 and 620 nm emission on an Envision (Perkin Elmer, Downers Grove, IL, USA). The TR-FRET 665 nm/620 nm ratio, which is inversely proportional to the production of cAMP, was used to determine the cAMP response. Non-specific binding was assessed using 100 μM non-labeled cAMP. The data were analyzed using the program PRISM to yield the EC_50_ and E_max_.

### 3.5. Chemistry

In order to find and characterize new ligands for the melatonin receptors, the following strategy was used. Positive ligands bearing either an iodine or bromide atom were selected from either our chemical series or from the vast HTS campaigns we conducted, In the case of bromide, the cold iodinated compound was synthesized and tested for its characteristics at the receptors. With these results in hand, the most interesting compounds were selected based on properties such as affinity (nanomolar range), MT_1_*versus* MT_2_ selectivity (specificity at MT1 *versus* MT2 receptors should be at least 2 logs in order to be usable as specific ligands, though no clear consensus exists on this particular point), functionality (agonists *versus* antagonists), and accessibility to the radio-iodination process.

### 3.6. Synthesis of Tert-butyl 2-(2-[(2-bromo-4,5-dimethoxyphenyl)methyl]-4,5-dimethoxy phenyl) Acetate (DIV879)

#### 3.6.1. General Procedures

All reactions were performed under a nitrogen atmosphere. Chemical reagents were purchased from classical suppliers and used without further purification. Thin-layer chromatography was conducted on silica gel plates pre-coated with aluminum (Macherey Nagel, Alugram^©^ SIG G/UV254, Düren, Germany). Column chromatography was performed using silica gel 60 (Macherey Nagel, 43–60 μm). NMR spectra were measured with a Bruker AV300 spectrometer (300 MHz for 1H, 75.5 MHz for ^13^C). Proton chemical shifts were referenced to CHCl_3_ (^1^H δ7.26, ^13^C δ77.0) in CDCl_3_. Mass spectrometry-coupled liquid chromatography analyses were carried out using a Waters Alliance 2695 apparatus (PDA 2996 detector and ZQ2000 Micromass, Waters, Milford, MA, USA).

DIV879 was discovered during a large high-throughput screening campaign as an MT_2_ antagonist with at least 2 logs poorer affinity for MT_1_. This compound bears a bromide.

#### 3.6.2. Synthesis of 6,7-dimethoxy-3-isochromanone (Compound **1**, Figure 3)

Ten milliliters of formaldehyde (37% in water) was added drop-wise to a solution of 10.3 g (52.4 mmol) 3,4-dimethoxyphenylacetic acid in a mixture of 10 mL of HCl 37% and 30 mL of acetic acid at room temperature. The mixture was heated to 90 °C over 1 h, cooled to room temperature, and hydrolyzed by the addition of 300 mL of water. The aqueous layer was extracted three times with dichloromethane. The organic layer was washed three times with saturated sodium bicarbonate solution, dried over magnesium sulfate, filtered, and evaporated to dryness to obtain 7.39 g of crude material. Trituration in isopropylic ether for 1 h resulted in 6.51 g (60%) of compound **1** as an off-white solid after filtration. 1H NMR (CDCl_3_) δ6.76 (s, 1H), 6.73 (s, 1H), 5.28 (s, 2H), 3.91 (s, 3H), 3.90 (s, 3H), 3.66 (s, 2H).

#### 3.6.3. Synthesis of 2-([(2-bromo-4,5-dimethoxyphenyl)methyl]-4,5-dimethoxyphenyl) Acetic Acid (Compound **2**, Figure 3)

A total of 2.3 g (10.8 mmol, 1.13 eq.) of bromoveratrole was added in one portion to a solution of 2.0 g (9.6 mmol) of 6,7-dimethoxy-3-isochromanone **1** in 20 mL of formic acid. The mixture was heated to 90 °C for 2 h and then hydrolyzed by the addition of 10 mL of water. The aqueous layer was extracted three times with ethyl acetate. The organic layer was washed twice with brine, dried over magnesium sulfate, filtered, and evaporated to dryness to yield 4.18 g of crude material. Trituration in a mixture of isopropylic ether/AcOEt resulted in 3.2 g (78%) of compound **2** as an off-white solid. ^1^H NMR (CDCl_3_) δ7.07 (s, 1H), 6.80 (s, 1H), 6.58 (s, 1H), 6.47 (s, 1H), 3.99 (s, 2H), 3.90 (s, 3H), 3.88 (s, 3H), 3.79 (s, 3H), 3.68 (s, 3H), 3.61 (s, 2H).

#### 3.6.4. Synthesis of Tert-butyl 2-(2-[(2-bromo-4,5-dimethoxyphenyl)methyl]-4,5-dimethoxy phenyl) Acetate (**DIV879**, Figure 3)

A total of 1.6 g (7.35 mmol, 3 eq.) *O*-tert-butyl-*N*,*N*′-diisopropylisourea was added in portions over a period of 2 h to a solution of 1.04 g (2.45 mmol) of acid **2** in 30 mL of dichloromethane at room temperature. The mixture was stirred overnight and filtered to remove insoluble materials. The filtrate was evaporated under vacuum and the residue purified twice by chromatography on silica gel (1-eluant: CH_2_Cl_2_/AcOEt 85/15, 2-eluant: heptane/AcOEt gradient) to obtain 749 mg (63%) of DIV879 as an off-white solid.

^1^H NMR (CDCl_3_) δ7.05 (s, 1H), 6.78 (s, 1H), 6.55 (s, 1H), 6.43 (s, 1H), 3.96 (s, 2H), 3.89 (s, 3H), 3.86 (s, 3H), 3.77 (s, 3H), 3.67 (s, 3H), 3.44 (s, 2H). ^13^C NMR (CDCl_3_) δ170.9, 148.4, 147.9, 147.3, 131.8, 130.2, 125.6, 115.3, 114.4, 113.6, 113.2, 113.1, 56.1, 55.8, 55.7, 39.5, 38.1, 27.9. LCMS (X-Bridge^©^, Waters, Milford, MA, USA, C18 4.6 × 150 mm, 5 μm) rt = 13.475 min (210.0 nm), UV > 98.7%; ESI [M + Na]^+^ = 503.3, 505.3.

### 3.7. Synthesis of DIV880

#### 3.7.1. Synthesis of 2-(2-[(3,4-dimethoxyphenyl)methyl]-4,5-dimethoxyphenyl) Acetic Acid (Compound **3**, Figure 4)

A total of 2.2 mL (17.3 mmol, 1.2 eq.) of veratrole was added to a solution of 3.0 g (14.4 mmol) of 6,7-dimethoxy-3-isochromanone 1 in 30 mL of formic acid at room temperature. The mixture was maintained at 95 °C overnight and then hydrolyzed by the addition of 30 mL of water. The aqueous layer was extracted three times with ethyl acetate. The organic layer was washed twice with brine, dried over magnesium sulfate, and evaporated to dryness to obtain 7.0 g of crude material. Chromatography on silica gel (eluant: heptane/AcOEt 1/1 + 1‰ AcOH) followed by trituration in isopropylic ether resulted in 2.2 g (44%) of compound 3 as an off-white solid after filtration. ^1^H NMR (CDCl_3_) δ6.75 (m, 2H), 6.62 (m, 3H), 3.91 (s, 2H), 3.86 (s, 3H), 3.83 (s, 3H), 3.78 (s, 3H), 3.77 (s, 3H), 3.55 (s, 2H).

#### 3.7.2. Synthesis of 2-(2-[(3,4-dimethoxyphenyl)methyl]-4,5-dimethoxy phenyl) Acetate (Compound **4**, Figure 4)

A total of 6.1 g (28.6 mmol, 4.5 eq.) of *O*-tert-butyl-*N*,*N*′-diisopropylisourea was added drop-wise over a period of 2 h to a solution of 2.2 g (6.35 mmol) of compound 2 in 33 mL of dichloromethane at room temperature. The mixture was stirred overnight and filtered to remove insoluble materials. The filtrate was evaporated under vacuum and the residue purified by chromatography on silica gel (eluant: heptane/AcOEt 7/3) to obtain 1.72 g (67%) of compound 3 as a yellow oil. ^1^H NMR (CDCl_3_) δ6.79 (d, 1H, J = 7.35 Hz), 6.78 (s, 1H), 6.64 (m, 3H), 3.93 (s, 2H), 3.89 (s, 3H), 3.87 (s, 3H), 3.83 (s, 3H), 3.81 (s, 3H), 3.46 (s, 2H).

#### 3.7.3. Synthesis of 2-(2-[(2-iodo-4,5-dimethoxyphenyl)methyl]-4,5-dimethoxy phenyl) Acetate (**DIV880**, Figure 4)

A total of 1.7 g (4.2 mmol, 1 eq.) of compound 2 dissolved in 25 mL of acetic acid was added drop-wise to a solution of 1.77 g (6.3 mmol, 1.5 eq.) of chloramine T and 944 mg (6.3 mmol, 1.5 eq.) of sodium iodide in 25 mL of acetic acid stirred over 1 h. After two hours at room temperature, the mixture was poured into 20 mL of water and extracted three times with ethyl acetate. The organic layer was washed twice with brine, dried over magnesium sulfate, and evaporated to dryness to yield 4.0 g of crude brownish material. Purification by chromatography on silica gel (eluant: heptane/AcOEt 8/2) resulted in 1.2 g (55%) of compound DIV880 as a yellow oil. ^1^H NMR (CDCl_3_) δ7.29 (s, 1H), 6.81 (s, 1H), 6.54 (s, 1H), 6.43 (s, 1H), 3.94 (s, 2H), 3.91 (s, 3H), 3.88 (s, 3H), 3.79 (s, 3H), 3.68 (s, 3H), 3.45 (s, 2H), 1.44 (s, 9H). ^13^C-NMR ( (CDCl_3_) δ 170.9, 149.5, 148.0, 147.4, 135.7, 130.5, 125.5, 121.6, 113.9, 113.5, 112.8, 88.7, 80.7, 56.1, 55.8, 55.8, 55.7, 43.2, 39.6, 28.0. LCUV (XTerra© MS C18 5 μm) rt = 13.476 min (285.5 nm) UV > 99.8%. ESI [M + Na]^+^ = 551.35.

### 3.8. Synthesis of SD6

#### 3.8.1. Synthesis of *N*-[2-(5-methoxy-1H-indol-3-yl)ethyl]iodoacetamide (SD6), Route A

**Figure 5:**
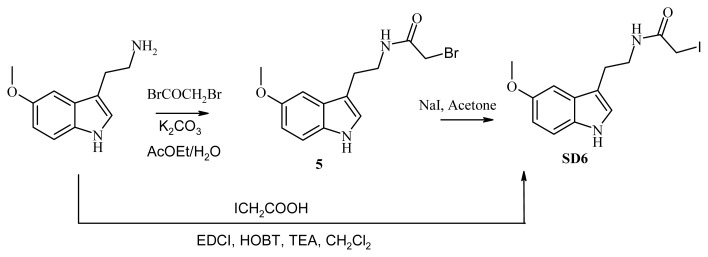
Schematic representation of the synthesis of SD6. (A) by reaction of 5-methoxytryptamine with bromoacetyl bromide in a biphasic medium (EtOAc-H2O) according to a variant of the Schotten–Baumann reaction (K_2_CO_3_) to obtain a bromoacetyl derivative [18]. Substitution of the bromine atom of compound **5** by refluxing in acetone with sodium iodide resulted in the iodo derivative **SD6**. (B) by a peptide-coupling reaction with iodoacetic acid in the presence of EDCI and HOBt in the presence of TEA in methylene chloride to generate the iodo derivative **SD6**.

*N-[2-(5-methoxy-1H-indol-3-yl)ethyl]iodoacetamide* (compound **SD6**, Figure 5) was obtained through two routes according to the synthetic pathway illustrated in Figure 5:

#### 3.8.2. Synthesis of *N*-[2-(5-methoxy-1H-indol-3-yl)ethyl]iodoacetamide (compound **SD6**, Figure 5), Route B

Method A: A solution of 3.11 g (0.01 mol) of compound **5** in 50 mL of anhydrous acetone was treated with 1.5 g (0.01 mol) of sodium iodide and the mixture heated at reflux for 2 h. After cooling, the reaction mixture was filtered and evaporated. The residue was then crystallized from toluene, resulting in 2.5 g (70%) of compound **5**.

Method B: A solution of iodoacetic acid (1.85 g, 0.01 mol) in 50 mL of methylene chloride was stirred at −10 °C for 20 min. Triethylamine (1.62 mL, 0.012 mol), EDCI (1.86 g, 0.0012 mol), and HOBt (1.62 g, 0.0012 mol) were added, and the mixture stirred at –10 °C for 30 min. A solution of 5-methoxy tryptamine (1.9 g, 0.01 mol) in 10 mL of methylene chloride was cooled at –10 °C and added drop-wise. After 6 h of stirring at room temperature, the reaction mixture was washed with water, a 1M HCl solution, water, a 10% NaOH solution, and water until a pH of 7 was reached. The organic phase was dried over MgSO_4_, filtered, and concentrated under reduced pressure. The residue was then crystallized from toluene, obtaining 2.6 g (73%) of **SD6**. Mp 159 °C; ^1^H NMR (80 MHz, CDCl_3_) δ8.32 (br s, 1H), 7.32 (d, 1H, *J* = 8.2 Hz ), 6.96 (d, 1H, *J* = 2.5Hz), 7.02 (d, 1H, *J* = 2.3 Hz), 6.80 (dd, 1H, *J* = 2.5 Hz and 8.2 Hz), 5.75 (br s, lH), 3.86 (s, 3H), 3.62 (m, 2H), 3.55 (s, 2H), 2.94 (t, 2H, *J* = 6.82 Hz). Anal. (C_13_H_15_IN_2_O_2_) C, H, N.

### 3.9. Synthesis of S70254

#### 3.9.1. Synthesis of 2-[5-methoxy-2-(naphthalen-1-yl)-1H-pyrrolo[3,2-b]pyridine-3-yl]ethan-1-amine (compound **8**, Figure 6)

**Figure 6:**
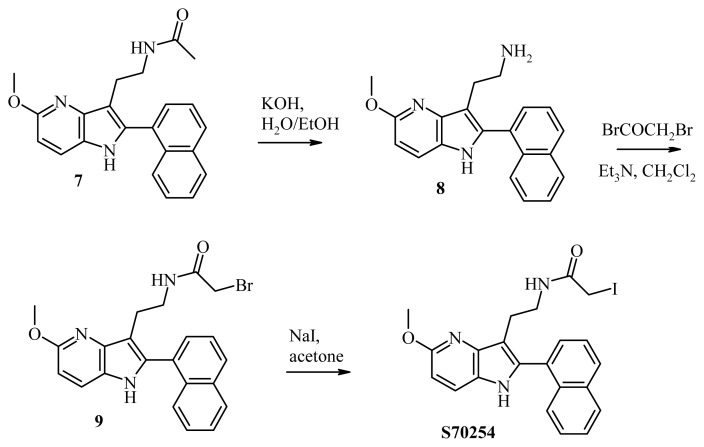
Schematic representation of the synthesis of S72054.

N-Acylatedazaindole (compound **7**, Figure 6) [19,20] (1.21 g, 3.4 mmol) was solubilized in a 1:10 water/methanol mixture. Potassium hydroxide (6.61 g, 118 mmol) was added and the reaction mixture refluxed for 87 h. After cooling, methanol was evaporated under reduced pressure and water (100 mL) added. The product was extracted with ethyl acetate (3 × 50 mL) and the organic phases combined and washed with a saturated aqueous solution of ammonium chloride (50 mL). The organic phase was dried over magnesium sulfate, filtrated, and evaporated under reduced pressure. The crude product was purified by silica gel column chromatography (ethyl acetate 84/methanol 15/ammoniac 1) to obtain amine **8** as a colorless oil (0.62 g).

Yield: 58%; ^1^H NMR (CDCl_3_; 250 MHz): δ8.45 (br s, 1H), 7.92–7.88 (m, 2H), 7.73 (d, *J =* 8.25 Hz, 1H), 7.58 (d, *J =* 8.75 Hz, 1H), 7.53–7.39 (m, 4H), 6.64 (d, 1H), 4.00 (s, 3H), 3.37 (br s, 2H), 3.00 (t, *J =* 6 Hz, 2H), 2.84 (t, *J =* 6 Hz, 2H); IR ν (neat, cm^−1^): 2,934, 1,613, 1,576, 1,242, 777; HRMS (ESI): calcd. for C_20_H_20_N_3_O [M+H]^+^ 318.160089; found 318.160154.

#### 3.9.2. Synthesis of 2-bromo-*N*-2-[5-methoxy-2-(naphthalen-1-yl)-1H-pyrrolo[3,2-b]pyridine-3-yl] Acetamide (Compound **9**, Figure 6)

A mixture of triethylamine (290 μL; 2.08 mmol) and amine **8** (600 mg; 1.90 mmol) in dichloromethane (20 mL) was cooled at −10 °C. A solution of bromoacetyl bromide (180 μL; 2.08 mmol) in dichloromethane (5 mL) was added and the reaction mixture stirred for 1 h. The resulting solution was washed with water (20 mL), and the organic phase dried over MgSO_4_ and evaporated under reduced pressure. The residue was purified by silica gel column chromatography (ethyl acetate 30/petroleum ether 70) to obtain 320 mg of compound **9** (S70253) as a white solid.

Yield: 59%; ^1^H NMR (CDCl_3_; 250 MHz): δ 8.18 (br s, 1H), 7.97–7.92 (m, 2H), 7.74 (d, *J =* 8.25 Hz, 1H), 7.63 (d, *J =* 8.75 Hz, 1H), 7.56–7.43 (m, 5H), 6.69 (d, *J =* 8.75 Hz, 1H), 4.09 (s, 3H), 3.60 (s, 2H), 3.57–3.52 (m, 2H), 2.93 (t, *J =* 6.25 Hz, 2H); Anal (C_22_H_20_BrN_3_O_2_)C, H, N; SM (ESI): m/z = 438 [M+H]^+^ (^79^Br), 440 [M+H]^+^ (^81^Br).

#### 3.9.3. Synthesis of 2-iodo-*N*-2-[5-methoxy-2-(naphthalen-1-yl)-1H-pyrrolo[3,2-b]pyridine-3-yl] Acetamide (**S70254**, Figure 6)

A mixture of sodium iodide (115 mg; 0.77 mmol) and bromo derivative **9** (310 mg; 0.7 mmol) in dry acetone (5 mL) was refluxed for 15 h. After cooling, the reaction was evaporated and dichloromethane (20 mL) added. The organic phase was washed with water (20 mL), dried over MgSO_4_, filtered, and evaporated under reduced pressure. The residue was purified by silica gel column chromatography (ethyl acetate 30/petroleum ether 70) to obtain 160 mg of the desired compound, **S70254**, after precipitation with ethyl acetate.

Yield: 47%; ^1^H NMR (CDCl_3_; 250 MHz): δ8.23 (br, 1H), 7.96–7.91 (m, 2H), 7.75–7.67 (m, 2H), 7.64 (d, *J =* 8.75 Hz, 1H), 7.58–7.43 (m, 4H), 6.70 (d, *J =* 8.75 Hz, 1H), 4.10 (s, 3H), 3.50–3.46 (m, 2H), 3.45 (s, 2H), 2.89 (t, *J =* 6.5 Hz, 2H); Anal (C_22_ H_20_ I N_3_ O_2_)C, H, N; SM (ESI): m/z = 486 [M+H]^+^.

### 3.10. Radio-Iodination

#### 3.10.1. Radio-Iodination of SD6 and S70254

Both radio-iodinated SD6 and radio-iodinated S70254 were synthesized by halogen exchange of their brominated precursors. Mixtures of Na^125^I (80.5 TBq/mmol) and brominated precursors were incubated for 12–15 h at ambient temperature. Carrier-free, mono-iodinated products were purified by HPLC.

#### 3.10.2. Radio-Iodination of DIV879

DIV879 was labeled with Na^125^I (80.5 TBq/mmol) using the chloramin1 T method [21]. The reaction was stopped with NaS_2_O_5_ and the carrier-free, mono-iodinated product purified by HPLC. The final compound, the iodinated analog of DIV879, was named DIV880.

### 3.11. Selectivity Studies for Melatonin Receptor Ligands

In order to evaluate ligand selectivity, the cold compound S70254, SD6, and DIV880 were submitted to our standard selectivity procedure. The specificity of the compounds was assessed by testing a standard set of receptors and a small number of enzymatic targets: (standard name of the receptor (species)/radioligand used for the experiments): NMDA(r)/[^3^H]-CGP 39653; AMPA(r)/[^3^H]-AMPA; A1(h)/[^3^H]-DPCPX; A2A(h)/[^3^H]-CGS 21680; α1(r)/[^3^H]-prazosin; α1A(h)/[^3^H]-prazosin; α1B(h)/[^3^H]-prazosin; α1D(h)/[^3^H]-prazosin; α2(r)/[^3^H]-RX 821002; α2A(h)/[^3^H]-RX 821002; α2B(h)/[^3^H]-RX 821002; α2C(h)/[^3^H]-RX 821002; β1(h)/[^3^H]-CGP 12177; β2(h)/[^3^H]-CGP 12177; Ca^2+^ Type L/[3H]-diltiazem; K^+^/ATP(r)/[^3^H]-glibenclamide; K^+^/VOLT(r)/[^125^I]-charybdotoxin; hERG1(h)/[^3^H]-dofetilide; muscarinic (r)/[^3^H]-QNB; σ(r)/[^3^H]-ditolylguanidine; dopamine D1(h)/[^3^H]-SCH 23390; dopamine D2(h)/[^3^H]-spiperone; GABA(r)/[^3^H]-GABA; histamine H1(h)/[^3^H]-pyrilamine; histamine H2(h)/[^125^I]-aminopotentidine; histamine H4(h)/[^3^H]-histamine; histamine H3(h)/[^125^I]-iodoproxyfan; I1p(b)/[^3^H]-clonidine; Y(r)/[^3^H]-neuropeptide Y; Nα4/β2(r)/[^3^H]-cytisine; N α4/β2(h); N alpha7(h); N α3/β2(h); PPARγ2(h)/[^3^H]-BRL 49653; OPIOID(r)/[^3^H]-naloxone; ET-A(h)/[^125^I]-endothelin 1; serotonin transporter(h)/[^3^H]-paroxetin; dopamine transporter(h)/[^3^H]-GBR 12935; noradrenalin transporter(h)/[^3^H]-nisoxetin; TP(TXA2/PGH2)(h)/[^3^H]-SQ29548; 5-HT2B(h)/[^3^H]-N-methyl-LSD; 5-HT(r)/[^3^H]-5-HT; 5-HT1A(h)/[^3^H]8-OH-DPAT; 5-HT2A(h)/[^125^I]-(±)DOI; 5-HT2A(h)/[^3^H]-ketanserin; 5-HT3(h)/[^3^H]-BRL 43694; 5-HT1B(h)/[^125^I]-CYP; 5-HT2C(h)/[^3^H]-mesulergine; 5-HT1D(h)/[^3^H]-serotonin; and MCH1(h)/[^125^I][Phe^13^,Tyr^19^]-MCH. Inhibition of the activity of the following enzymes was also tested: caspase-3(h); EGFR kinase(h); and PKCα(h). For all of the details and protocols, go to www.cerep.org. Our compounds did not exhibit any activity towards these targets; they were mainly inactive or barely active (less than 20% effect) at 10 μM. This margin was thought to be enough to consider the compounds specific for the melatonin receptor(s).

## 4. Conclusions

Though the description of SD6 did not result in a new tool for studying melatonin receptors, the present work permitted the discovery, synthesis, and characterization of two specific ligands for the MT2 isoform: S70254 and DIV880. Complete characterization of the kinetics of ligand binding has started in order to determine if the behavior of these two ligands is different from [^125^I]-2IMLT and [^3^H]-melatonin using the available melatonin receptors from various species [14,16,22,23]. The next steps will comprise a comparison of the pharmacology of these ligands using a rather large set of compounds already described by our group, as well as binding and autoradiography using native organ membrane preparations or slices. We are still searching for a MT_1_-specific ligand. Unfortunately, the melatonin receptor ligand literature is sparse in regards to compounds with specificity better than 2 logs between receptors, despite several attempts by others [24,25] and our own group [26–28].

## Figures and Tables

**Figure 3:**
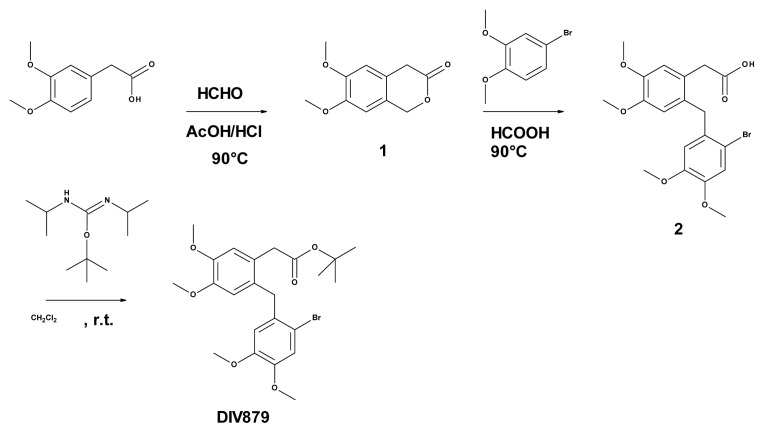
Schematic representation of the synthesis of DIV879.

**Figure 4:**
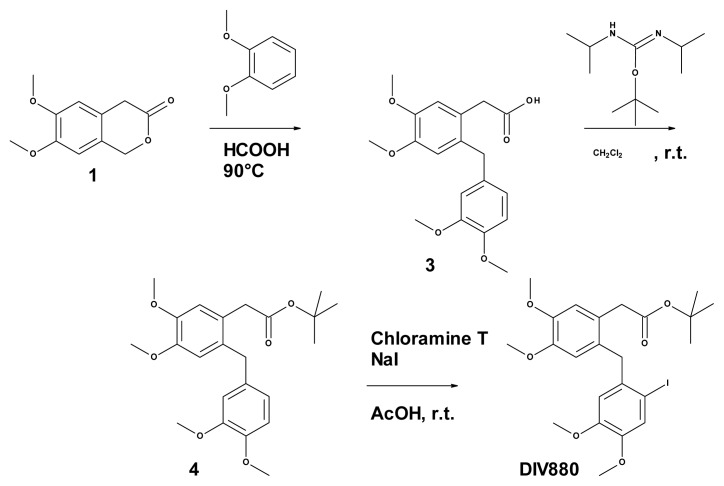
Schematic representation of the synthesis of DIV880.

**Table 1:** Affinity and functional constants of the candidates for new radioligands for melatonin receptors compared to 2-iodomelatonin (2IMLT). Data are mean ± SEM of at least three independent experiments. The [^35^S]-GTPγS binding assay results are presented as the percentage of the assay conducted under the same conditions using melatonin as the agonist and taken as 100%.

A—hMT_1_	Affinity	[^35^S]-GTPγS		TR-FRET-cAMP	
	
	pK_i_	pEC_50_	E_max (%)_	pEC_50_	E_max (%)_
2IMLT	10.44 ± 0.08	9.79 ± 0.11	108 ± 3	10.09 ± 0.01	90 ± 5
SD6	9.94 ± 0.01	9.79 ± 0.17	115 ± 10	8.58 ± 0.14	103 ± 10
S70254	6.18 ± 0.10	7.10 ± 0.04	15 ± 2	5.84 ± 0.14	78 ± 12
DIV879	6.25 ± 0.03	<5	ND	ND	ND
DIV880	6.08 ± 0.01	5.9 ± 0.02	10 ± 1	<5	ND

**B—hMT_2_**	**Affinity**	** [^35^S]-GTPγS**		**TR-FRET-cAMP**	
	
	**pK_i_**	**pEC_50_**	**E_max (%)_**	**pEC_50_**	**E_max (%)_**

2IMLT	9.80 ± 0.05	9.80 ± 0.12	121 ± 13	10.15 ± 0.002	99 ± 2
SD6	9.89 ± 0.22	9.97 ± 0.05	114 ± 16	9.16 ± 0.02	103 ± 1
S70254	8.73 ± 0.23	8.69 ± 0.30	43 ± 1	7.47 ± 0.21	76.5 ± 1
DIV879	8.14 ± 0.04	7.91 ± 0.161	58 ± 2	ND	ND
DIV880	8.02 ± 0.02	7.97 ± 0.18	67 ± 8	7.79 ± 0.09	97 ± 1

**Table 2:** pK_d_ and B_max_ values for radioligands of the MT_1_ and MT_2_ receptors. Data are mean ± SEM of at least three independent experiments.

	[^125^I]-2IMLT		[^125^I]-SD6		[^125^I]-S70254		[^125^I]-DIV880	
	
	pK_d_	B_max_ fmol/mg of protein	pK_d_	B_max_ fmol/mg of protein	pK_d_	B_max_ fmol/mg of protein	pK_d_	B_max_ fmol/mg of protein
hMT_1_	10.69 ± 0.07	688 ± 153	10.85 ± 0.01	276 ± 50	-	-	-	-
hMT_2_	10.16 ± 0.03	1,998 ± 318	10.18 ± 0.11	1,929 ± 308	9.61 ± 0.14	1,778 ± 87	9.65 ± 0.07	2,308 ± 0.07
